# Historical contingency and productivity effects on food-chain length

**DOI:** 10.1038/s42003-019-0287-8

**Published:** 2019-01-28

**Authors:** Hideyuki Doi, Helmut Hillebrand

**Affiliations:** 1 Institute for Chemistry and Biology of the Marine Environment, Carl-von-Ossietzky University Oldenburg, Schleusenstrasse 1, 26382 Wilhelmshaven, Germany; 2 Graduate School of Simulation Studies, University of Hyogo, 7-1-28 Minatojima-minamimachi, Chuo-ku, Kobe, 650-0047 Japan; 3 Helmholtz-Institute for Functional Marine Biodiversity at the University Oldenburg (HIFMB), Ammerländer Heerstrasse 231, 26129 Oldenburg, Germany; 4 Alfred-Wegener Institute, Helmholtz Centre for Polar and Marine Research, Bremerhaven, 27570 Germany

## Abstract

Food-chain length (FCL) is a fundamental ecosystem attribute, integrating information on both food web composition and ecosystem processes. It remains untested whether FCL also reflects the history of community assembly known to affect community composition and ecosystem functioning. Here, we performed microcosm experiments with a copepod (top predator), two ciliate species (intermediate consumers), and bacteria (producers), and modified the sequence of species introduction into the microcosm at four productivity levels to jointly test the effects of historical contingency and productivity on FCL. FCL increased when the top predator was introduced last; thus, the trophic position of the copepod reflected assembly history. A shorter FCL occurred at the highest productivity level, probably because the predator switched to feeding at the lower trophic levels because of the abundant basal resource. Thus, we present empirical evidence that FCL was determined by historical contingency, likely caused by priority effects, and by productivity.

## Abstract (web summary)

Hideyuki Doi and Helmut Hillebrand investigate how the sequence of species immigration affects food-chain length and whether the effect depends on productivity. Using a microcosm experiment including a copepod predator, ciliate species, and bacteria, they find that food chains are longest when the top predator is added last and that high productivity reduces food-chain length.

## Introduction

Food-chain length (FCL), a measure of the number of trophic levels in a system^1–4^, is a property of food web structure with connection to community composition^1–3^ and ecosystem processes, such as energy and matter flows in ecosystems^2,5^, and CO_2_ exchange between freshwater systems and the atmosphere^6^. Also, FCL determines the level and timing of bioaccumulation of potentially toxic substances in food webs and thus indirectly relates to human health^7,8^. Thus FCL has been recognized as a fundamental ecosystem attribute and has been extensively studied^7–11^, although the question of which factors constrain FCL still remains under debate.

Numerous hypotheses on constraints of FCL have been proposed and are widely cited^1–3,9–11^. Among these studies, a majority focus on a few general hypotheses such as the productivity (basal resource availability), ecosystem size, and disturbance hypotheses^12–14^. The productivity hypothesis predicts that FCL increases with increasing productivity, because higher energy availability at the base of the food web allows for the existence of higher trophic levels, given the transfer efficiencies between trophic levels^2,11,15–17^. However, Kondoh and Ninomiya^15^ suggested that FCL could be shorter with increasing productivity when adaptively foraging predators switch their diet to a more basal resource and thus to a lower trophic position. No change in, or shortening of, FCL^16^, with enrichment of productivity, can also occur if food web structure changes because of different functional responses^17^ or if productivity relates to higher instability of the community^18^. The ecosystem size hypothesis predicts that FCL increases with increasing ecosystem size, such as lake volume^12^. The disturbance hypothesis, also termed as the dynamic constraints hypothesis, predicts that more frequent or more intense disturbance in ecosystems would shorten FCL, because longer chains are less resilient and thus unlikely to persist in disturbed habitats^13^. Still, among the common FCL hypotheses, the productivity hypothesis has been tested most frequently but with incongruent results from field and laboratory studies^13,14,19,20^.

Recently, nitrogen stable isotope measurements have become the technique most often used for FCL determination, next to gut content analyses^12–15,20,21^. Nitrogen stable isotope composition reflects the trophic position of consumers^22^. Nitrogen isotopes provide a measure of realized FCL, integrating the assimilation of energy or mass flow through all the trophic pathways leading to top predators^22^.

In large lakes, FCL was shown to increase in older lakes, probably indicating that the ecosystem’s history of species immigration and evolution affected FCL^23^. The effect of species immigration history on FCL suggests that colonization sequence may affect community structure^24^. Historical contingency has frequently been considered in ecology^24^ in the context of community structure, species diversification, and productivity–diversity relationships^24–29^. Despite the accepted role of historical contingency with regard to community composition and ecosystem functioning^24–28^, its importance as a determinant of FCL has, to our knowledge, never been tested.

Here we provide an experimental test of historical contingency on FCL, specifically colonization order, along a gradient of productivity using microcosm experiments. We find that FCL is determined by historical contingency, via priority effects and productivity.

## Results

### FCL of the microcosm systems

We combined four colonization sequences and four productivity levels in a fully factorial design with five replicates (80 microcosms in total). Such replicated microcosm experiments have been widely used to test hypotheses in ecology and evolutionary biology^30^. The productivity gradient was established by protozoan pellet concentration. We inoculated our microcosms with bacteria, ciliates, and zooplankton, allowing for a food web structure including bacteria as a basal resource, a primary consumer (the bacterivore ciliate *Tetrahymena* sp., abbreviated T), an intra-guild predator (the bacterivore/intra-guild predator ciliate *Blepharisma* sp., abbreviated B), and a copepod (*Cyclops* sp., abbreviated C) as the top predator. The groups were added in four specific sequences into the microcosm at each of four productivity levels. Then we measured the stable nitrogen isotope of the top predator zooplankton to estimate the FCLs in the microcosms. We also evaluated the abundance and body mass of the species to show the shifts in community structure in the microcosms.

**Fig. 1:**
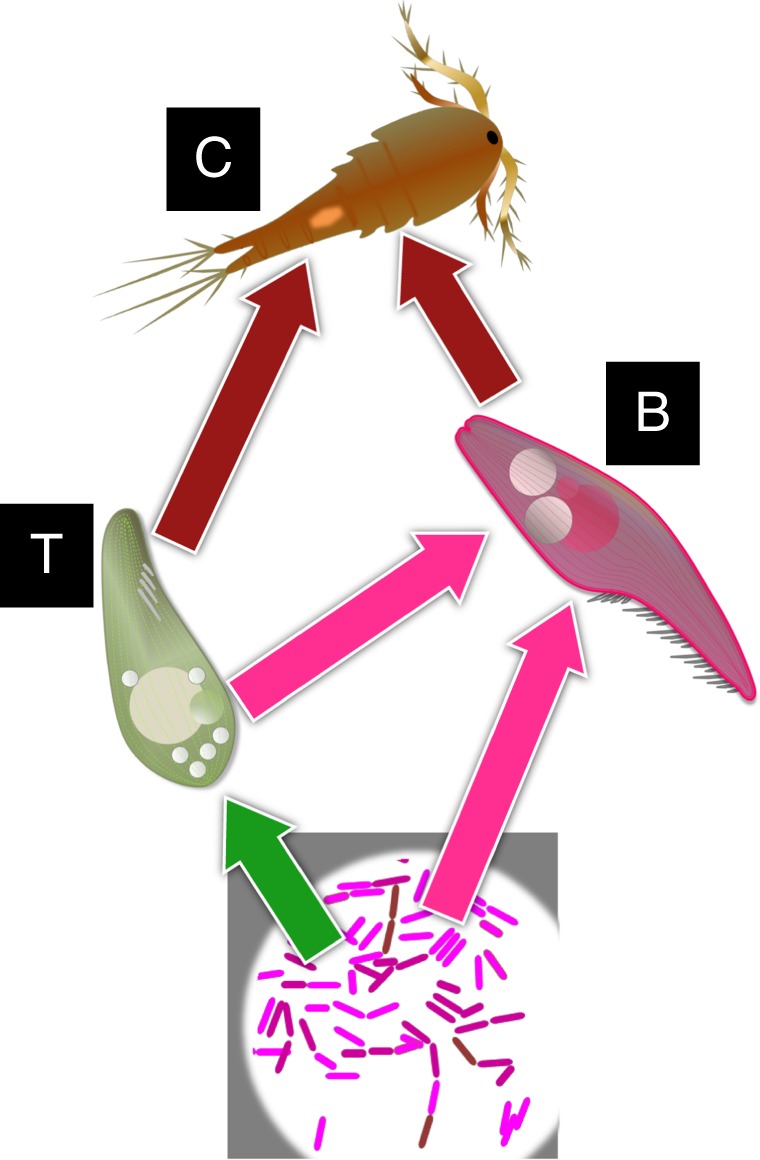
Illustration for the food webs in the microcosm experiment. The letters (B, T, and C) indicate *Tetrahymena* sp., *Blepharisma* sp., and *Cyclops* sp., respectively. The arrows indicate link of food web with regard to our preliminary experiments and previous studies

**Fig. 2:**
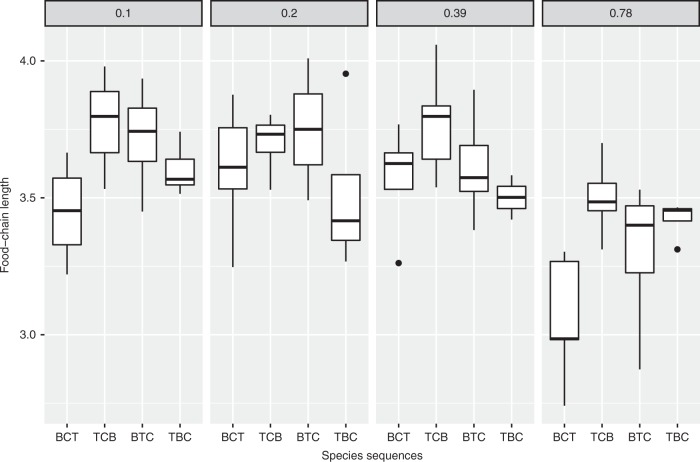
The food-chain lengths (FCLs) with different sequence treatments (named in Table 1) and productivity from 0.1 to 0.78 g pellet L^−1^ (*N* = 5 for each treatment). The boxes indicate ± 25% quartiles with the median (bar), and the bars indicate ± 1.5× quartiles. The points are outliers

With the results from microcosm experiment (Fig. 1), the calculated FCLs remarkably varied with productivity and species sequences (BCT, TCB, BTC, TBC, see Table 1; Fig. 2), reflected by significant main effects of the factor in the general linear model (GLM; Table 2). These effects were independent, as the interaction was not significant (Table 2). The sequences BTC and TBC, at which copepods were introduced last, had significantly higher FCLs than sequences with earlier introduction of the copepod (Table 2). The differences in FCLs correspond to a shift of 0.5 trophic level in these treatments, indicating that the trophic position of the top predator was substantially changed. Thus later entry by the top predator led to a more vertical organization of the food web. At the highest productivity (0.78 g pellet L^−1^), the FCLs were significantly shorter than at the other productivity level (Table 2).

**Table 1:** The introduction sequences for the FCL experiments

Treatment name	First sequence	Second sequence	Third sequence
BCT	*Blepharisma* (B)	*Cyclops* copepod (C)	*Tetrahymena* (T)
TCB	*Tetrahymena* (T)	*Cyclops* copepod (C)	*Blepharisma* (B)
BTC	*Blepharisma* (B)	*Tetrahymena* (T)	*Cyclops* copepod (C)
TBC	*Tetrahymena* (T)	*Blepharisma* (B)	*Cyclops* copepod (C)

**Table 2:** Results of GLM for sequence (BCT, TCB, BTC, and TBC) and productivity (0.1, 0.2, 0.39, and 0.78 g L^−1^ of protozoan pellets) effects on FCLs

Factors	*t* Value	*p* Value	Comparisons
Sequence	2.05	0.045	0.1, 0.2, 0.39: BTC > TBC > TCB = BCT 0.78: BTC > TBC = TCB = BCT
Productivity	–2.85	0.006	0.78 *<* 0.1, 0.2, 0.39
Sequence×productivity	–1.26	0.212	

The comparisons means significant difference (*α* = 0.05) by Turkey multiple comparisons among the treatments

### Abundance and body mass of species

**Fig. 3:**
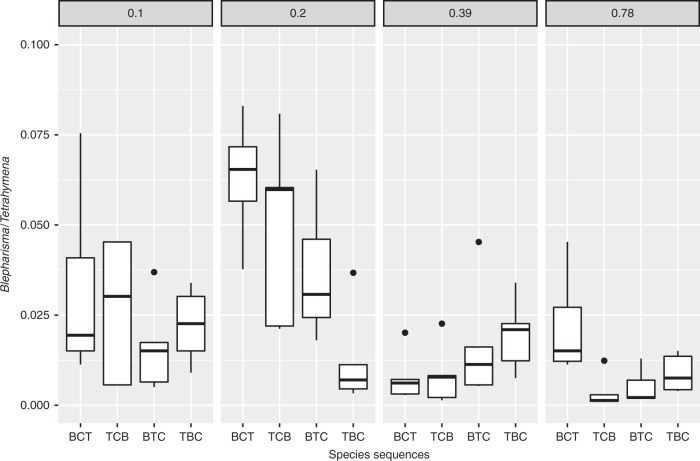
The abundance ratio of *Blepharisma*/*Tetrahymena* on different productivity from 0.1 to 0.78 and sequence treatments (named in Table 1, *N* = 5 for each treatment). The boxes mean ± quartiles with median (point), and the bars indicate ± 1.5× quartiles (*N* = 5)

The abundance of ciliate consumers varied among productivity levels and introduction sequences (Supplementary Fig. 1 and 2, Supplementary Table 1). The abundance of *Tetrahymena* was not different between the sequences, thus the food source for both intraguild predator and top predator probably was not limiting during the experiment. However, the abundance of *Tetrahymena* significantly increased with productivity (Supplementary Table 1). The abundance ratio of *Blepharisma*/*Tetrahymena* varied correspondingly not only between productivity levels but also with introduction sequences. The ratios were higher in the BTC sequence, where *Blepharisma* was introduced before *Tetrahymena* (Fig. 3). The individual body mass (Supplementary Fig. 3) and the number of surviving individuals (mean = 23.4 ± 0.5 individuals at the final experimental day) of copepods were not significantly different between the treatments (Supplementary Table 1). The survival rates of copepods were very high (94% remained) such that the predator population was almost completely maintained to the final day of the experiment.

## Discussion

FCL varied between 3 and 4 with species inoculation sequence and productivity in our microcosm systems. In natural systems, FCL generally ranges between 3 and 6^13,14^. The effect of introducing the copepod last corresponded to an increase in FCL by 0.5 trophic positions, whereas the FCL reduction in the highest productivity level corresponded to 0.3–0.4 trophic positions. Thus our microcosms provided first evidence for a variability of FCL with community assembly history and novel insights in the relationship between productivity and FCL.

When the top predator invaded in the food web later, it performed as a top predator mainly feeding on the intraguild predator (*Blepharisma*), and consequently the system had a longer FCL. When the top predator copepods were introduced before the intraguild predator (*Blepharisma*), they only fed on the intraguild prey (*Tetrahymena*) feeding on bacteria, thereby depressing the population level of *Tetrahymena*. Therefore, *Blepharisma* had a trophic level of ~2, similar to *Tetrahymena*. The copepod, whether it fed on *Tetrahymena* or *Blepharisma*, would then have a trophic level of ~3.

When *Blepharisma* primarily feeds on *Tetrahymena*, the copepod fed on a prey with a trophic level >2. Therefore, another mechanism can be assumed that the intraguild predator did not successfully compete for the bacteria with the intraguild prey and consequently had lower abundances. Moreover, lower *Blepharisma*/*Tetrahymena* ratios were observed at TCB sequence, which may also reduce the trophic position of the top predator due to lowered relative abundance of the intraguild predator. Our experiment therefore highlights the role of priority effects of consumer introduction and top predator’s adaptive foraging for defining maximum FCL. We found such priority effects on FCL in a reduced web with three consumer species only, therefore it will be important to analyze historical contingencies in real ecosystems, in order to see whether our results can be transferred to natural ecosystems with more complex network structures.

Productivity is generally supposed to lengthen FCL in aquatic systems^13,14^, but in this study, at the highest productivity, the FCL were shorter. The food-web model by Kondoh and Ninomiya^15^ suggested that FCL can be shorter with increasing productivity when considering adaptive foraging of consumers. This requires that some of the predators are generalists able to feed on different food sources. Predators may switch from higher trophic levels to lower ones at higher productivity if especially the basal species becomes more abundant^31,32^. In fact, the abundance of the primary consumer *Tetrahymena* increased at highest productivity, which may explain the shortened FCL we found at high productivity levels, if the top predator fed more on the more abundant primary consumer, *Tetrahymena*. We have no evidence whether the intraguild predator (*Blepharisma*) also changed its foraging to bacteria with productivity as we did not measure the isotopic composition of the ciliates in our experiment. Some of the previous studies reported the lack of a positive correlation between productivity and FCL^12,14,21^, which could be explained by historical contingency masking the productivity effect on FCL. If the adaptive foraging of top predator changed the food web structure along with historical contingency, the predator would maximize the food-web stability according to the expectation by the mathematical model of Kondoh^33^. Also, the adaptive foraging of top predator in food web may minimize the destabilizing effects of productivity enrichment in natural habitats^34^. We did not directly test such changes^35^, but see such analyses as potential future advance to more fundamentally understand the historical contingency effects on food web structure.

In conclusion, we obtained evidence from microcosms that FCL varies with historical contingency of community assembly and productivity of system. If these results from a small-scale experimental study prove to be valid in more complex natural systems, these results represent an initial step to understand the lasting impact of food-web assembly on food-web structure in an immigration context.

## Methods

### Microcosm experiment

We used a two-way factorial design with four productivity levels and four species-introduction sequences (Table 1) as treatments. Each of the 16 unique treatment combinations (4 productivity levels×4 sequences) was established in five replicates, totaling 80 microcosms.

As microcosms, we used 250-mL Pyrex glass flasks, filled with 100 mL of medium. The different productivity levels were established by different concentrations of protozoan pellets (Carolina Biological Supply [CBS], Burlington, NC, USA): 0.1, 0.2, 0.39, and 0.78 g L^−1^ of protozoan pellets were added to natural spring water (Volvic, from Clairvic Spring, Auvergne Regional Park, France). With regards to the previous microcosm studies^27,36^, we set the weight of protozoan pellets for the gradient of productivity levels. Flasks with medium were autoclaved and then inoculated with the basal producer in the form of four bacteria cultures (*Bacillus subtilis*, *Bacillus cereus*, *Proteus vulgaris*, *Serratia marcescens*, from CBS).

The bacteria were allowed to grow for 7 days before we added the other species in four sequences (Table 1). These species comprised two ciliates (*Tetrahymena* sp., *Blepharisma* sp.) and a copepod (*Cyclops* sp.). *Tetrahymena* is a bacterivore^37^ and represented the primary consumer (Fig. 1), *Blepharisma* is an interguild predator able to feed on bacteria and *Tetrahymena*^38^, and *Cyclops* was added as top predator feeding on ciliates^39^. Also, *Cyclops* can adaptively alter their diets in experimental environments^39^. *Tetrahymena* and *Blepharisma* were provided by CBS, stocked in separate 250-mL flasks with a pellet-bacteria medium with 0.78 g L^−1^ of the protozoan pellets. *Cyclops* copepods were originally collected from an agricultural pond (34° 41’ 22” N,132° 73′ 71″ E, Higashi-Hiroshima, Japan) by towing a 250-µm meshed plankton net, picked up by a pipette under a binocular, and incubated with the above medium with *Tetrahymena* and 0.78 g pellet L^−1^.

From the six possible sequences of these three species, we established four sequences (Table 1), omitting those where the top predator would be introduced first without suitable food. The first, second, and third species were added in weekly intervals, 7, 14, and 21 days after the bacteria inoculation. We introduced 35 of the copepods and 100 individuals of each ciliate species by using a pipette. For copepods, we filtered the incubated medium by 250-µm mesh and then rinsed with natural spring water to reduce the contamination with the ciliates. By rinsing of the copepods, we can reduce the ciliate contamination. We picked the copepods from the mesh by a pipette and transferred them into microcosms. For ciliates, 0.5 mL of the ciliate stocking cultures were sampled just before inoculation to estimate population densities by a ×400 microscope.

The microcosms were run as semicontinuous batch cultures. We renewed 10% of medium once a week, i.e., during each introduction timing. We mixed the microcosms and replaced 10 mL (10%) with fresh medium of the same productivity level^23,35^. During the FCL experiment, all 80 microcosms were situated in an incubator (KCSLPH-1400CT, Nippon Medical & Chemical instruments Co. Ltd., Osaka, Japan) at 20 °C with a 12/12 light/dark cycle.

### Sampling and counting

After day 28 (21 days from first introduction), we collected the copepods. We filtered the medium on a glass filter (GF/F, GE Healthcare) and picked copepods under a binocular. We directly put copepods in predried and preweighted tip cups. The body mass of copepod was determined by weighing the individuals in their tip cup on a high-precision balance (BM-20, A&D, Tokyo, Japan).

The isotope samples of protozoan pellet and the tip cup with copepods were dried at 60 °C for 24 h and stored in a desiccator. The 0.5 mL of medium was collected for estimating population density of the ciliate species. The medium was fixed by 2% acidic Lugol’s solution, and population density was counted at ×400 magnification using a Leitz DMIL microscope.

### Stable isotope analysis

The nitrogen stable isotope (δ^15^N) of the samples were determined using a PDZ Europa ANCA-GSL elemental analyzer interfaced to a PDZ Europa 20-20 isotope ratio mass spectrometer (Sercon, Cheshire, UK) at Stable Isotope Facility of the University of California Davis. Nitrogen isotopic data are reported using the conventional δ notation, where δ^15^N = (^15^N/^14^N_sample_/^15^N/^14^N_standard_ − 1) × 1000 (‰). Air N_2_ were used as international standard for δ^15^N. We did not measure the δ^13^C of the samples due to limited sample mass.

### Calculation of FCL

FCL is defined as the trophic position of the top predator (copepod) in each microcosm. We assumed a trophic fractionation value of 3.4‰ to calculate FCL based on previous studies on food webs and FCLs^22,40^. The value has widely been used for FCL studies on metazoans, and also the trophic enrichment of ciliate was close to this general enrichment value (Supplementary Information, 3.6–3.7‰). Consequently, FCL was calculated as

$$2 + \frac{{{\mathrm{\delta}} ^{15}{\mathrm N}_{{\mathrm{copepod}}} - {\mathrm{mean}\,{\mathrm\delta}} ^{15}{\mathrm N}_{{\mathrm{pellet}}}}}{{3.4}}$$

where, the mean δ^15^N_pellet_ was −2.54 ± 0.3‰ (Supplementary Information, *n* = 6, mean ± 1 SD).

Before the experiment, we tested the isotope turnover time for copepods by comparing samples 7 and 14 days after introducing the copepod to the same mixture of ciliates and bacteria. As the result, the nitrogen stable isotope values saturated at days 7 and 14 after introduction (see Supplementary Information), similar to the previous study using small invertebrates^41–44^. From a model for the relationships between half-life days of isotope turnover and invertebrate body size, which was provided by a meta-analysis^45^, the isotopic half-life for the copepods can be predicted to be 2.21 ± 0.39 days (mean ± 95% confidence interval, see Supplementary Information). Thus, by this calculation, the isotopic turnover time of the copepod is fast enough to calculate the trophic position (i.e., FCL of the system) after 7 days (see the result of supplemental experiments in the Supplementary Information, Supplementary Figs. 4–8). Simple time-dependent differences in the copepods’ isotope values are not expected to alter the FCL data from the species-sequencing experiment. Also, we used the same population of copepods for this study, thus, we do not expect isotope differences in initial copepods.

### Statistical analysis

We performed GLMs for evaluating the effects of colonization sequence and productivity on FCL, the abundance/biomass of the different species, and the ratio of *Blepharisma*/*Tetrahymena*. The error distribution was set as Gaussian distribution in general but negative binomial for the ratio of *Blepharisma*/*Tetrahymena*. To detect significant differences between treatment levels, we performed Turkey multiple comparisons for significant treatments. Statistical significance was set at *α* = 0.05, and all analyses were performed using R 3.3.1^46^ with ggplot2 and glm packages for graphics and GLMs, respectively. All the data are available in Dryad (10.5061/dryad.2m9r762).

## Supplementary Information

Supplementary Information

## Data Availability

All data, including the abundance of species and FCL in this study, are available via the Dryad Digital Repository (10.5061/dryad.2m9r762).
