# Supplementary material for: Historical contingency and productivity effects on food-chain length
Source: Commun Biol. 2019 Jan 28;2:40. doi: 10.1038/s42003-019-0287-8 (PMC6349908; doi:10.1038/s42003-019-0287-8)
Supplement: Supplementary file 1 — Supplementary Information [file 42003_2019_287_MOESM1_ESM.pdf]

Supplementary Materials

Supplementary Method for preliminary experiments for estimating trophic enrichment factor of ciliate and isotope changes in copepod

We performed the experiments to estimate the trophic enrichment factor of ciliate and isotope changes in copepod for calculating the FCLs in the mesocosms.

The microcosms were conducted by 250-mL Pyrex glass flask filled with 100 mL of medium with different productivity. The medium with 0.78 g of the protozoan pellet (Carolina Biological Supply (CBS), Burlington, North Carolina, USA) in 1-L natural spring water (Volvic, from Clairvic Spring, Auvergne Regional Park, France). Then, the flasks with medium were autoclaved. Then, we inoculated the medium with four bacteria species (*Bacillus cereus, Bacillus subtilis, Proteus vulgaris, Serratia marcescens*, from CBS). We distributed the medium to microcosms for a week after bacteria inoculation.

We used two ciliate species; *Tetrahymena* sp., and *Blepharisma* sp. and *Cyclops* copepods for the experiment. We set three experiments; 1) *Tetrahymena* only and 2) *Blepharisma* only, and 3) *Tetrahymena* and *Cyclops* copepods, with triplicates. We introduced the species by after a week of bacteria inoculation with triplicates of above microcosms. We introduced 35 and 100 individuals of copepods and ciliate species by using a pipette, respectively. For ciliate, just before introduction, the 0.5-mL of the ciliate stocking cultures were sampled to estimate population densities by a x400 microscope to determine volumes for the introduction corresponding to 100 individuals per microcosm. To standardize the initial number of individuals of each species, if necessary, by diluting them before introductions. For experiments 1 and 2, at day 14, we

filtered the medium by the glass filter (GF/F, GE Healthcare), and concentrated the ciliate on the filter. Also, for experiment 3, the samples at the initial population, day 7 and 14 , we collected picked up copepod under a binocular and directly put in the tip cup.

The protozoan pellet and above samples was dried at 60°C for 24 h and stored in a desiccator. The nitrogen stable isotope ($\delta^{15}N$) of the samples were determined using a PDZ Europa ANCA-GSL elemental analyzer interfaced to a PDZ Europa 20-20 isotope ratio mass spectrometer (Sercon, Cheshire, UK) at Stable Isotope Facility of the University of California Davis. Nitrogen isotopic data are reported using the conventional δ notation, where $\delta^{15}N = (^{15}N/^{14}N_{sample}/^{15}N/^{14}N_{standard} - 1) \times 1000$ (‰). Air N_2 were used as international standard for $\delta^{15}N$.

As the results of Exps. 1 and 2, the mean $\delta^{15}N$ of pellet, *Tetrahymena*, and *Blepharisma* were –2.54 ± 0.3‰ (n = 6, mean ±1SD), 1.09 ± 0.1‰ (n = 3), and 1.15 ± 0.4‰ (n = 3), respectively, so trophic enrichment from the pellet ($\delta^{15}N_{ciliate}$ – mean $\delta^{15}N_{pellet}$ (–2.54)) to *Tetrahymena* and *Blepharisma* was 3.6 ± 0.1 and 3.7± 0.4‰, respectively.

For Exp. 3, the $\delta^{15}N$ of copepods were not significantly different between days 7 (4.79 ± 0.87‰, mean ±1SD) and 14 (4.70 ± 0.70‰) (Tukey multiple comparison, $p > 0.05$, n = 3), and significantly different from the initial value (3.57 ±0.48‰) and days 3 (3.82 ± 0.59‰), Tukey multiple comparison, $p = 0.012$, Fig.P1). From these results, we assumed that the effect of introduction timing on the copepods' isotope values can be ignored in the microcosm experiment.

In fact, the previous experimental studies for isotope turnover suggested 3-5 days for the saturations of isotopic changes in small macroinvertebrates, including zooplankton, and chironomid larvae (Doi et al. 2007, Gamboa-Delgado and Le Vay

2008; Larsen et al. 2011; Overmyer et al. 2008). In addition, a meta-analysis study by Vander Zanden et al. (2015) provided a linear model to predict the species-specific half-life time of isotope turnover using the organisms' body size. From the model, the isotopic half-life time of the copepods can be expected to be 2.21 ± 0.39 days (mean ±95%CI). This again is much faster than the 7-day period we chose for our experiment.

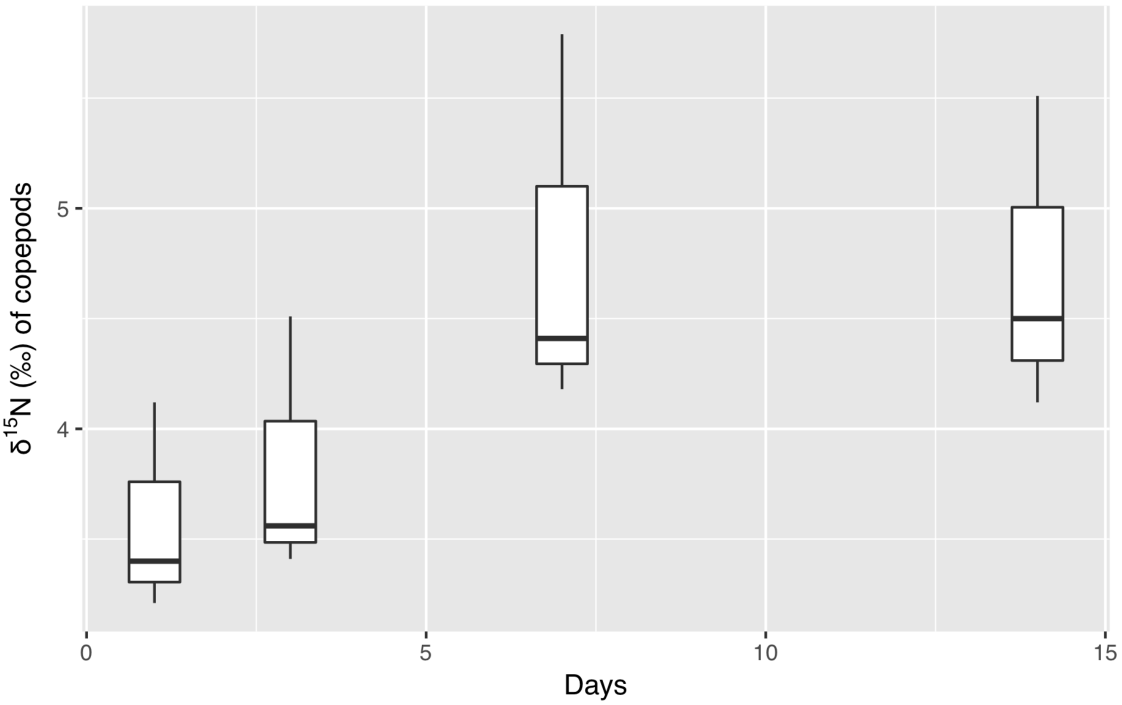

Supplementary Figure 1 The time-series data for $\delta^{15}N$ of copepods in the preliminary experiment. The boxes indicate ± 25% quartiles with the median (bar), and the bars indicate ± 1.5x quartiles.

References

Doi, H., Kikuchi, E., Takagi, S., & Shikano, S. Changes in carbon and nitrogen stable isotopes of chironomid larvae during growth, starvation and metamorphosis. *Rapid Comm. Mass Spec.*, **21,** 997-1002 (2007).

Gamboa-Delgado, J., Le Vay, L. Natural stable isotopes as indicators of the

relative contribution of soy protein and fish meal to tissue growth in Pacific white shrimp (*Litopenaeus vannamei*) fed compound diets. *Aquaculture,* **291,**115-123 (2009).

Larsen, T., Ventura, M., O'Brien, D. M., Magid, J., Lomstein, B. A., & Larsen, J. Contrasting effects of nitrogen limitation and amino acid imbalance on carbon and nitrogen turnover in three species of Collembola. *Soil Biol. Biochem.,* **43,** 749-759 (2011).

Overmyer, J. P., MacNeil, M. A., & Fisk, A. T. Fractionation and metabolic turnover of carbon and nitrogen stable isotopes in black fly larvae. *Rapid Comm. Mass Spec.,* **22,** 694-700 (2008).

Vander Zanden, M. J., Clayton, M. K., Moody, E. K., Solomon, C. T., & Weidel, B. C. Stable isotope turnover and half-life in animal tissues: A literature synthesis. *PLoS ONE,* **10,** e0116182 (2015).

Supplementary Method for preliminary experiments for estimating population dynamics in the microcosm experiment.

We performed the experiments to confirm the stability in the population dynamics of the ciliate and copepod species in the microcosm for evaluating the population dynamics.

The microcosms were conducted by 250-mL Pyrex glass flask filled with 100 mL of medium with different productivity. The different productivity levels were established by different concentration of protozoan pellet (Carolina Biological Supply [CBS], Burlington, North Carolina, USA): 0.1, 0.2, 0.39 and 0.78 g L^{-1} of protozoan pellet were added to natural spring water (Volvic, from Clairvic Spring, Auvergne Regional Park, France). Then, the flasks with medium were autoclaved. Then, we inoculated the medium with four bacteria species (*Bacillus cereus, Bacillus subtilis, Proteus vulgaris, Serratia marcescens*, from CBS). We distributed the medium to microcosms for a week after bacteria inoculation.

We used two ciliate species; *Tetrahymena* sp., and *Blepharisma* sp. and *Cyclops* copepods for the experiment at the same time. We set four productivity treatments with gradient of pellet content (0.1, 0.2, 0.39, and 0.78 g L^{-1}) with four replicates. We introduced the species by after a week of bacteria inoculation with triplicates of above microcosms. We introduced 35 and 100 individuals of copepods and ciliate species by using a pipette, respectively (Note: the initial individuals of copepods was 30 in the FCL experiment). For ciliate, just before introduction, the 0.5-mL of the ciliate stocking cultures were sampled to estimate population densities by a x400 microscope to determine volumes for the introduction corresponding to 100 individuals

per microcosm. We standardized the initial number of individuals of each species by diluting them before introductions, if necessary. In days 1, 2, 3, 4, 5, 6, 7, 10, 14 ,18, 0.5-mL of the ciliate stocking cultures were sampled to estimate population densities of the ciliates by a x400 microscope.

As the results of Exp. 2, the abundance fluctuation in three species microcosms seems to be stable after 5 days in the both ciliate species (Fig. P2 and P3). The treads were general in four productivity gradients, although the timings of the abundance peaks were different among the gradients. Also, the ratios of the ciliate species were also seems to be stable after 5-7 days (Fig. P4). The results would suggest that the FCL experiment can be conducted within 5-days inculcation after final introduction in the microcosm system. Thus, we collected the final samples in the FCL experiment after 7 days from final introduction of the species in the microcosms.

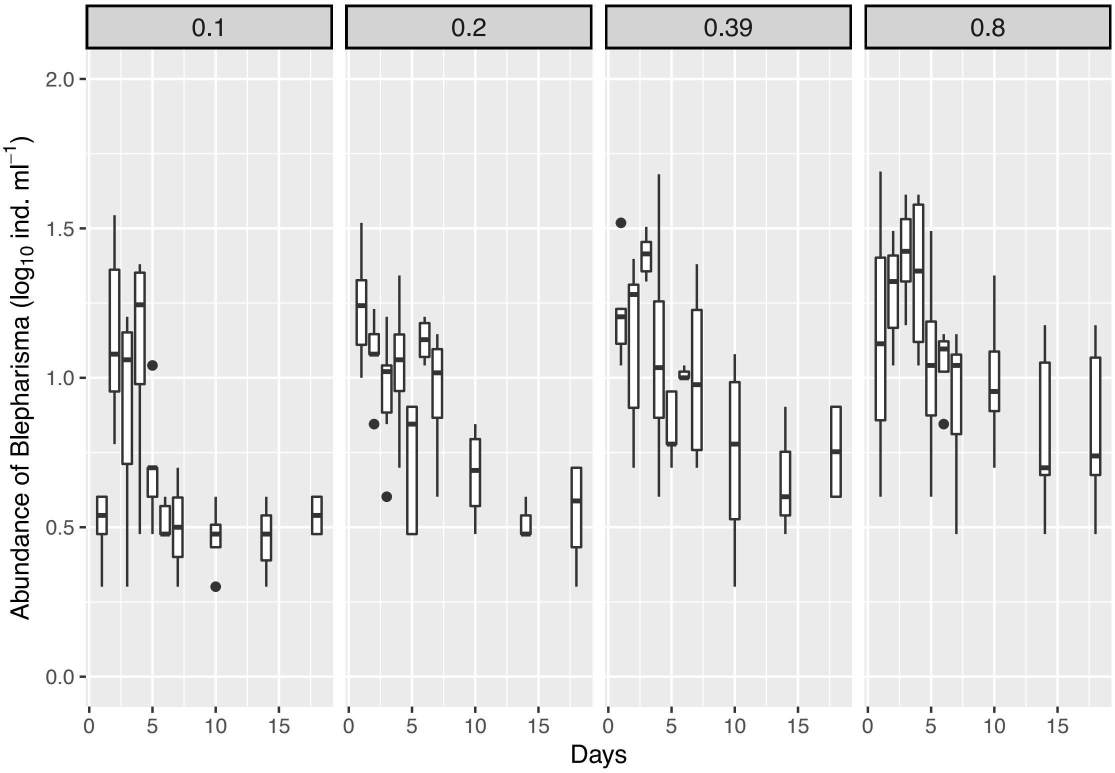

Supplementary Figure 2 The time-series plot in abundance of *Blepharisma* on different productivity from 0.1 to 0.78 and sequence treatment from preliminary Exp. 3. The boxes indicate ± 25% quartiles with the median (bar), and the bars indicate ± 1.5x quartiles. The points are outliners.

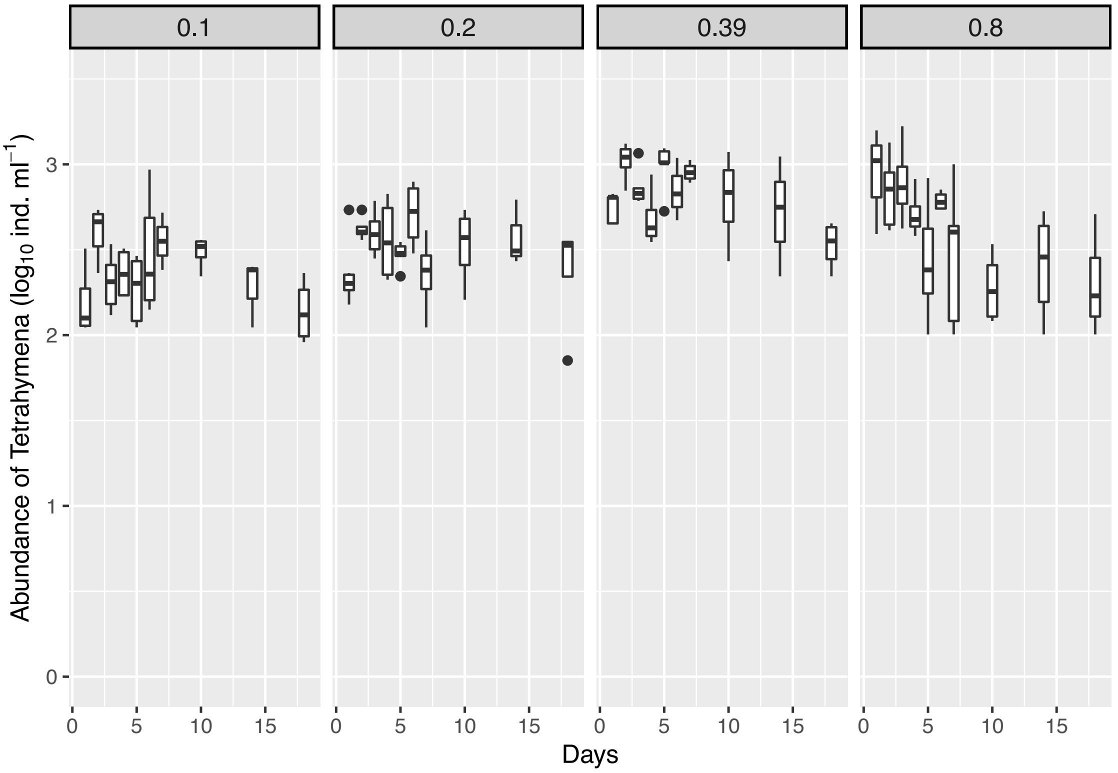

Supplementary Figure 3 The time-series plot in abundance of *Tetrahymena* on different productivity from 0.1 to 0.78 and sequence treatment from preliminary Exp. 3. The boxes indicate ± 25% quartiles with the median (bar), and the bars indicate ± 1.5x quartiles. The points are outliners.

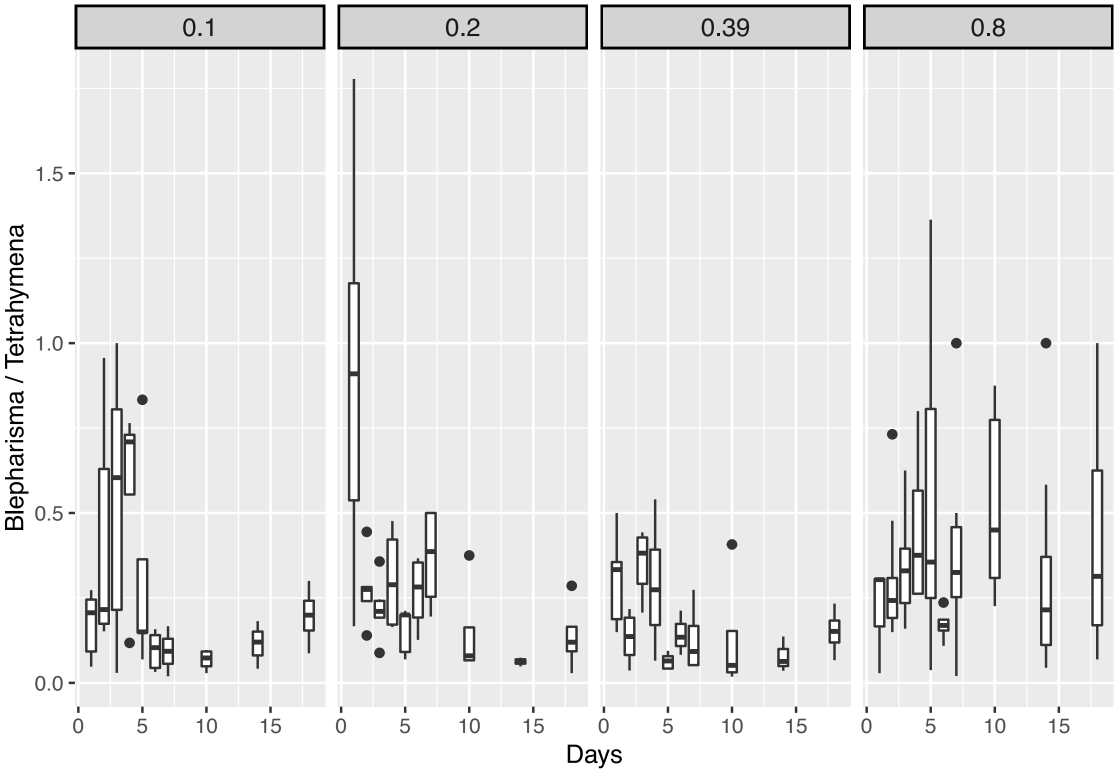

Supplementary Figure 4 The abundance ratio of *Blepharisma* / *Tetrahymena* on different productivity from 0.1 to 0.78 and sequence treatment in preliminary Exp. 3. The boxes mean± quartiles with median (point), and the bars indicate ± 1.5x quartiles (*N* =5).

Supplementary Table 1 The results of GLMs and Turkey multiple comparisons for sequence and productivity effects on abundance of ciliates (a,b), the abundance ratio (c), body mass of copepod (d) and the number of survival individuals (e). The comparisons by Turkey test means significant difference (α=0.05) in the sequence and productivity treatments.

a) Abundance of *Blepharisma*

Factors	F value	p-value	Comparisons
Sequence	-2.135	0.03658	BTC < Others
Productivity	4.200	<0.001	0.2 > Others
Sequence*Productivity	-2.996	0.00389	

b) Abundance of *Tetrahymena*

Factors	t value	p-value	Comparisons
Sequence	-0.999	0.321563	
Productivity	3.787	<0.001	0.78>0.39 >0.2=0.1
Sequence*Productivity	2.636	0.010513	

c) Blepharisma / *Tetrahymena*

Factors	t value	p-value	Comparisons
Sequence	-1.272	0.207852	BTC > Others
Productivity	4.257	<0.001	0.2>Others
Sequence*Productivity	-3.54	<0.001	

d) Mean body mass of copepod

Factors	t value	p-value	Comparisons
Sequence	1.192	0.238	
Nutrient	1.201	0.234	
Sequence*Nutrient	0.941	0.35	

e) The number of survival individuals copepod

Factors	t value	p-value	Comparisons
Sequence	0.50	0.602	
Nutrient	–0.02	0.876	
Sequence*Nutrient	1.221	0.220	

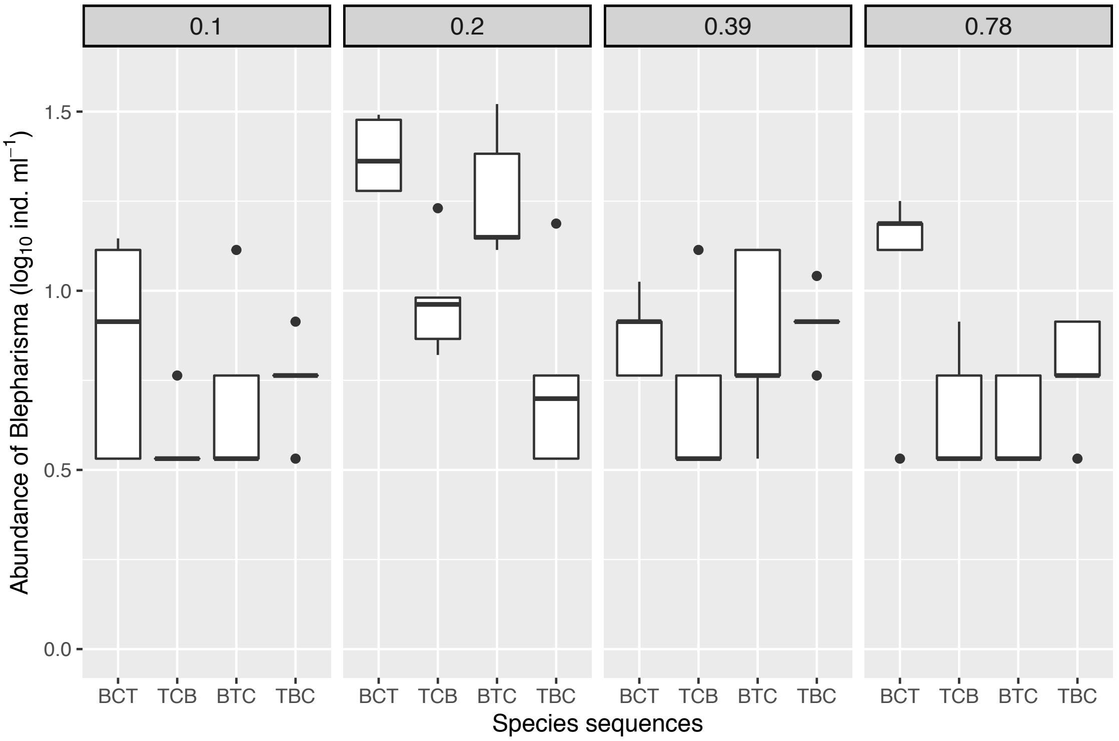

Supplementary Figure 5 The abundance of *Blepharisma* on different productivity from 0.1 to 0.78 and sequence treatments (named in Table 1, *N* =5 for each treatment). The boxes mean ± quartiles with median (point), and the bars indicate ± 1.5x quartiles.

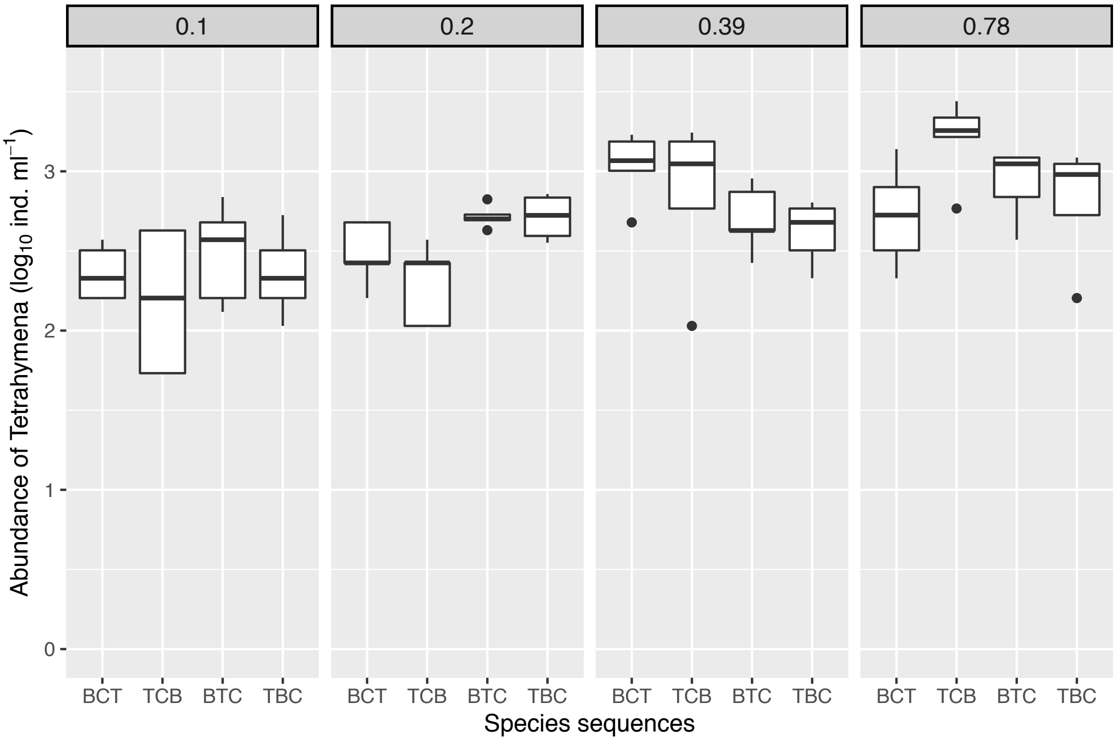

Supplementary Figure 6 The abundance of *Tetrahymena* on different productivity from 0.1 to 0.78 and sequence treatments (named in Table 1, N =5 for each treatment). The boxes mean ± quartiles with median (point), and the bars indicate ± 1.5x quartiles.

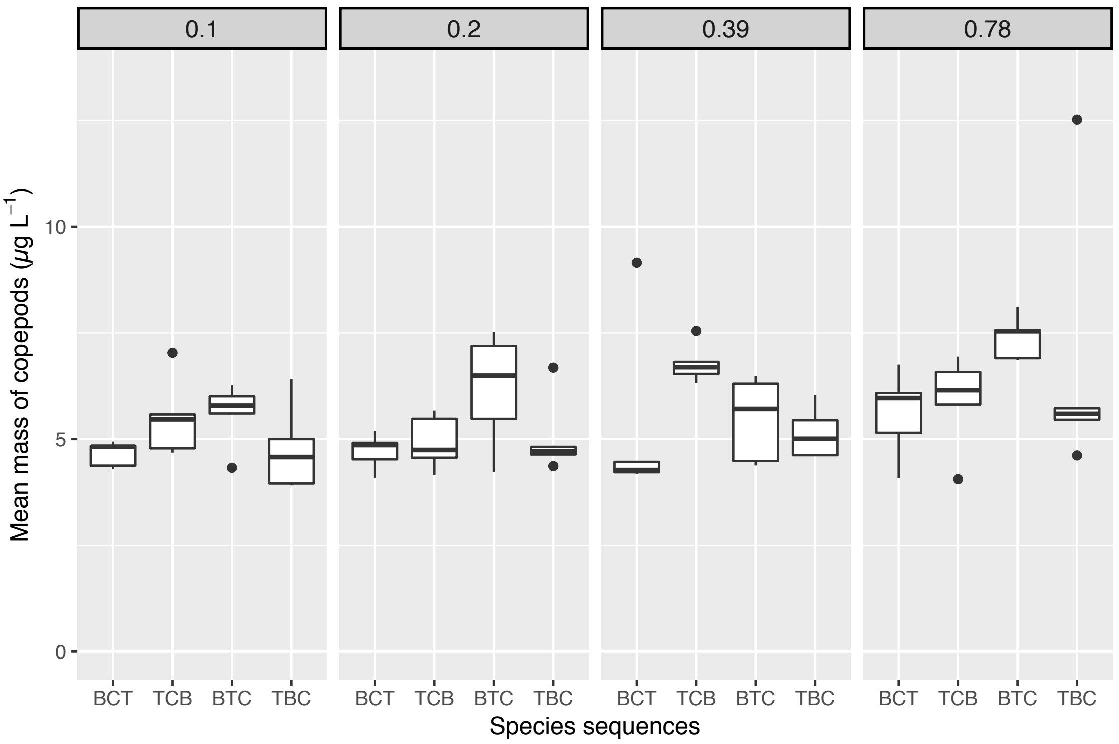

Supplementary Figure 7 The mean body mass of copepods in each microcosm on different productivity from 0.1 to 0.78 and sequence treatments (named in Table 1, *N* =5 for each treatment). The boxes mean the ± quartiles with median (point), and the bars indicate ± 1.5x quartiles.

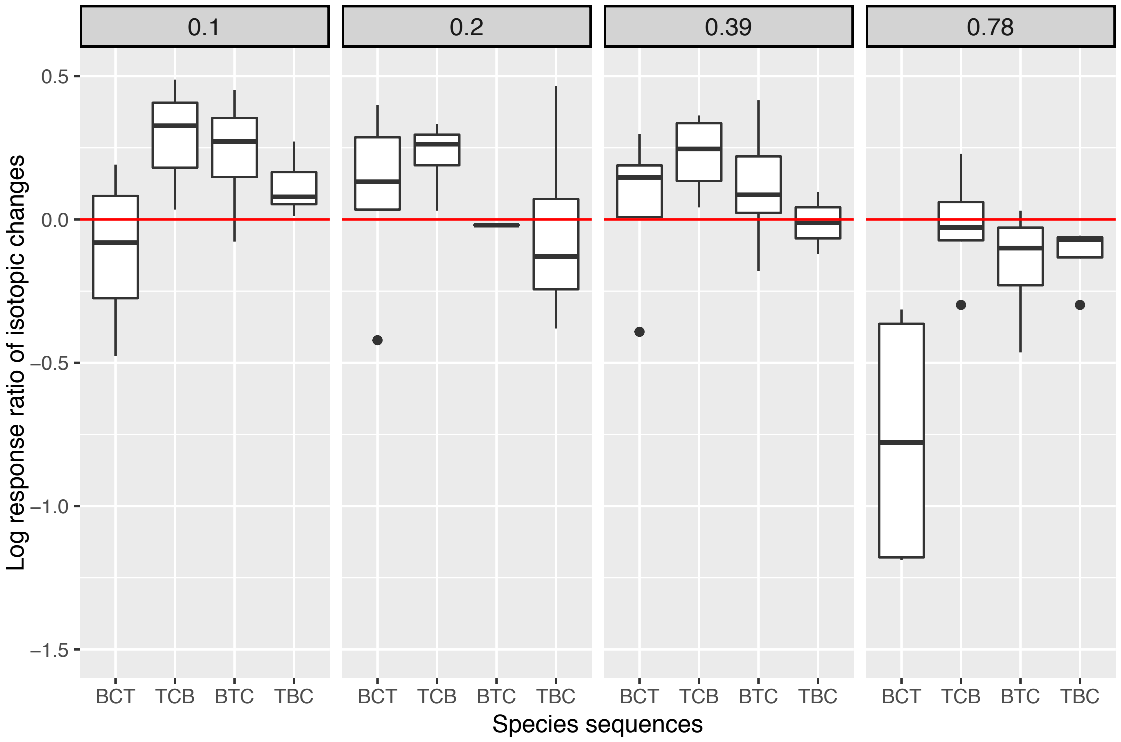

Supplementary Figure 8 The log response ratio of $\delta^{15}N$ values changes in the copepods (Ln (measured/initial copepod $\delta^{15}N$ values) on different productivity from 0.1 to 0.78 and sequence treatments (named in Table 1, *N* =5 for each treatment). The boxes mean the ± quartiles with median (point), and the bars indicate ± 1.5x quartiles. The red line indicates the zero log-response ratio, indicating non-significant effect size of isotopic changes.
